# The Secret Out

**Published:** 1861-05

**Authors:** 


**THE SECRET OUT.**—A very imprudent physician has done his brethren a great injury by thoughtlessly divulging one of the most valuable secrets of the profession, while riding up to Union Square in the Fourth Avenue cars yesterday. "How is practice now? you must be making a great deal of money, for every third person seems to be ailing?" "True; there is much serious sickness, but I get no practice. Secession has made the times so hard, that people cure themselves by eating nothing."

There are a few bodily ailments which are aggravated, and in some cases rendered incurable by insufficient diet; but with the exception of Diphtheria and a few others, nine out of ten of all ordinary ailments are controlled, are arrested, are permanently cured by a wise diminution of the amount of food eaten. This is particularly the case when there is no decided ailment, but a general feeling of discomfort or of unwellness. In all actively inflammatory maladies, where there is acute pain any where, total abstinence from all substantial food, from every thing liquid or solid, except hot teas, is the sheet-anchor of safety, when not extended beyond thirty-six hours. No one should venture on a longer abstinence on any occasion without the advice of a physician.

All pain is caused by over-distended blood-vessels pressing against some neighboring nerve. Hence the quickest way of relieving any ordinary pain is to diminish the amount of blood in the vessels of the part by bleeding. But there is a safer, a better and a more enduring relief in cutting off the supply of blood; and as blood is made out of the food we eat, it must be apparent, that if on the feeling of pain or discomfort, we cease eating absolutely, that pain must begin to diminish within six hours, that being the time required for converting food into blood, and if no more food is eaten, no more blood can be made; while the amount in the system is diminished at the rate of two or more pounds in every twenty-four hours of invalidism, there must be relief. But let it be remembered that while this diminution goes on in a state of rest by means of the perspiration, sensible and insensible, as well as by all the involuntary motions of the system, and the friction of the blood along its vessels, it is an important fact that every crook of the finger, every wink of the eye, every thought of the mind, is at the expense of the consumption of a greater or less number of particles of the body; so that, in every succeeding moment, the body weighs less than it did the preceding moment; this diminution takes place in the amount of the circulating fluids directly. If then the slightest motion diminishes the amount of blood, and it is the excess of blood in a part that causes pain, the next best means of diminishing pain, after cutting off the supply of food, is exercise. Hence the more a man exercises *short of actual fatigue*, the better he will feel, the sooner and more effectually will he be relieved. Many a time a man has felt uncomfortable, sometimes very decidedly so, but upon taking a walk or ride, or engaging in some interesting work, he expresses himself as having been greatly relieved. Let then this thought impress itself on the mind, that in the common every day ailments of life we must look for the cause in an excess of blood and other fluids in the body, and that whatever diminishes that excess is curative. The methods of this diminution are worth remembering. 1. Abstinence from food. 2. Perspiration, whether induced by covering up in bed and drinking hot teas, or by muscular exercise. 3. Vomiting. 4. Bleeding. 5. Counter-irritation, as by frictions with the hand or a mustard-plaster. Of these we recommend only abstinence, perspiration, exercise, friction. But let it be remembered that when exercise evidently increases the discomfort or the pain, or when it induces a positive feeling of weariness, it is contra-indicated, and quiet of mind and repose of body should be sedulously cultivated. And under all circumstances let it be remembered that however beneficial exercise may be in any given case, the very moment it becomes a felt fatigue, that moment it becomes a positive injury, if persisted in.

